# Relative Efficacy of Commercially Available Ultraviolet-C Devices in the United States With Sufficient Irradiation Dose Delivery to Horizontal Surfaces

**DOI:** 10.7759/cureus.105184

**Published:** 2026-03-13

**Authors:** Stephanie Gibbons, Franklin Dexter, Randy W Loftus, Melinda S Seering, Jonathan E Charnin

**Affiliations:** 1 Anesthesiology, University of Iowa, Iowa City, USA; 2 Anesthesiology and Perioperative Medicine, Mayo Clinic, Rochester, USA

**Keywords:** delivery, horizontal surfaces, irradiation dose, pathogenic, staphylococcus aureus, ultraviolet-c, uv-c

## Abstract

Background: Improved environmental cleaning in the anesthesia workspace is indicated to reduce surgical site infections. We evaluated the time for delivery of a mean (standard deviation) of 27 (0.15) mJ/cm^2^, the minimally effective irradiation dose required for attenuation of *Staphylococcus aureus* multilocus sequence type 5, to radiometers positioned horizontally on the anesthesia machine tray and medication cart using commonly employed ultraviolet-C (UV-C) technology.

Methods: This was a multicenter, controlled (by standardized equipment positioning) simulation study evaluating triangular (Helios, Surfacide, Waukesha, WI 53186) and single emitter (Tru-D, Memphis, TN 38104, and Xenex *San Antonio, TX 78216*) UV-C configurations. A series of standardized UV-C treatment trials was completed for each device. Following manufacturer recommendations and typical use, treatment duration was five minutes for Xenex and up to 30 minutes for Helios and Tru-D devices. The primary outcome was the cumulative maximal irradiation dose delivered to calibrated radiometers positioned horizontally on the anesthesia machine and cart. The trials were fully independent replications.

Results: Xenex achieved 27 mJ/cm^2^ at the anesthesia machine and cart for 1/16 trials and 0/16 trials, respectively. TruD achieved a total of 27 mJ/cm^2^ at the anesthesia machine within five minutes for 16/16 trials and at the anesthesia cart within 30 minutes for 0/16 trials. Helios achieved 27 mJ/cm^2^ at the anesthesia machine for 4/8 trials by five minutes and 8/8 trials by 16.2 (3.10) minutes and at the anesthesia cart for 0/8 trials within five minutes and 8/8 trials within 16.2 (3.10) minutes. The proportion of trials achieving the target dose differed significantly between devices (0/16 for Xenex, 0/16 for Tru-D, and 8/8 for Helios). Pairwise comparisons showed that Helios performed significantly better than both other devices (adjusted P < 0.0001 for each comparison).

Conclusion: The Helios device can deliver the minimally effective dose of 27 mJ/cm^2^ to horizontal surfaces within a 30-minute treatment period, which is feasible for the perioperative arena.

## Introduction

Surgical site infections (SSIs) are associated with increased patient morbidity and mortality [1] and affect up to 11% of patients undergoing open surgery [2]. SSIs prolong hospital admission and increase the risk of intensive care unit (ICU) stay [3], hospital readmission, and healthcare costs [4]. Further work is indicated to enhance SSI prevention.

Pathogen transmission among anesthesia workspace reservoirs (e.g., from the anesthesia machine to the patient's nose after anesthesia is induced) is tightly associated with SSI development [5,6]. The risk of SSI is 2% without transmission of *Staphylococcus aureus* (*S. aureus)* and 18% with transmission of *S. aureus* isolates resistant to the prophylactic antibiotic [5]. Implementation of an evidence-based, multifaceted infection control program in the anesthesia workspace can generate substantial reductions in pathogen transmission and subsequent 90-day postoperative SSIs [6,7]. Improved cleaning of the anesthesia machine and equipment is an important component of the multifaceted approach [6-8].

*S. aureus* multilocus sequence type 5 (MLST 5) is associated with increased strength of biofilm formation, desiccation tolerance, and antibiotic resistance versus other *S. aureus* strains frequently isolated from anesthesia workspace reservoirs [9,10]. MLST 5 transmission occurring despite current surface disinfection cleaning procedures is associated with increased risk of SSI development [9-11]. Recent work has evaluated innovative UV-C treatment strategies designed to address this more pathogenic strain characteristic [12-14].

The minimally effective UV-C dose required to achieve a 6-log reduction in MLST 5 is a mean (standard deviation) of 27.01 (0.15) mJ/cm^2^ [12,13]. This dose can be practically and safely delivered by anesthesia consultants to vertical surfaces directly exposed to UV-C irradiation with two minutes of treatment using a triangular configuration of UV-C emitters (one emitter at the head of the surgical bed and an emitter on each side of the bed) [12,14]. While the triangular configuration is designed to ameliorate barriers to UV-C delivery, such as indirect exposure of horizontal surfaces [12-15], the time required for delivery of the minimally effective dose to horizontal surfaces in the anesthesia workspace has not yet been rigorously assessed [12]. In simulation, for nine radiometers positioned at 69.5 inches from the floor, nine feet from a triangular emitter configuration, and oriented horizontally to the emitters, the maximal irradiation dose was approximately 1.6 mJ/cm^2^ after a two-minute treatment period [12]. Thus, greater than two minutes of treatment will be required to address horizontal surfaces. Further, the energy delivered from widely used, alternative UV-C technology involving single emitters rotating 360 degrees to horizontal surfaces may be impacted by several barriers. These include distance from the emitter to target environmental surfaces, various heights from the floor, surface orientation to the emitters, and differences in surface material that may result in shadowing. Thus, dose delivery from single emitters in the presence of barriers should be assessed and compared to the triangular configuration, which is specifically designed to combat barriers, potentially resulting in shadowing [14]. These are important knowledge deficits that currently limit the reliable employment of available UV-C technology for attenuation of more pathogenic strain characteristics in the anesthesia workspace to improve patient safety [5,6,14].

In this study, our primary aim was to compare the time required for delivery of the minimally effective dose for *S. aureus *MLST 5 [9,10] attenuation to horizontal surfaces in the anesthesia workspace (anesthesia machine tray and cart) by widely utilized UV-C technology involving triangular (Helios, Surfacide, Waukesha, WI 53186) and single emitter (Tru-D, Memphis, TN 38104 and Xenex, San Antonio, TX 78216) configurations. The anesthesia cart, by containing a vertical surface facing the emitter and substantial height from the floor, provided an opportunity to assess the impact of shadowing with each of the devices. The total treatment time was limited to five minutes by Xenex technology because the machine cannot be adjusted to increase time and to 30 minutes for the Tru-D and Helios devices, given that 30 minutes is the maximum time considered practical for real-world use [16]. Our secondary aim was to compare ozone generation and inadvertent UV-C exposure among devices. These study results can guide strategic use of commonly employed UV-C technology available in the United States to attenuate more pathogenic *S. aureus* strain characteristics that are frequently transmitted from environmental surfaces in the anesthesia workspace and potentially involving surfaces oriented horizontally to UV-C emitters [9-15]. 

## Materials and methods

Overview

The Tru-D device was evaluated at the University of Iowa in Iowa City, IA, and the Xenex and Helios devices at the Mayo Clinic in Rochester, MN. At each site, a discussion with environmental services was held to ensure that devices were evaluated in a manner consistent with typical use and that device maintenance was according to manufacturer recommendations. 

Pilot study for anesthesia workspace equipment standardization

Ten operating rooms were randomly selected for observation at the University of Iowa following terminal cleaning. Anesthesia workspace equipment type and mean distance along X- and Y-axes from the center of the surgical bed were recorded and summarized (Table 1).

**Table 1:** Average distance of object surfaces from ten operating rooms compared to the center of the surgical bed (origin). x = horizontal axis from center of bed, y = vertical axis from center of bed, h = height from floor to first shelf on the anesthesia station, h2 = height from floor to second shelf on the anesthesia station

	x (inches)	y (inches)	h (inches)	h_2_ (inches)
Surgical bed	0	0	34.20	
Surgical table	-5.26	-129.24	39.08	
Anesthesia bag	3.19	65.49	35.10	
IV pole	-15.94	58.31	50.43	
Anesthesia station	0.36	90.34	33.48	60.84
Computer	10.29	98.44	44.78	
Anesthesia cart	55.49	119.76	43.28	

**Figure 1:**
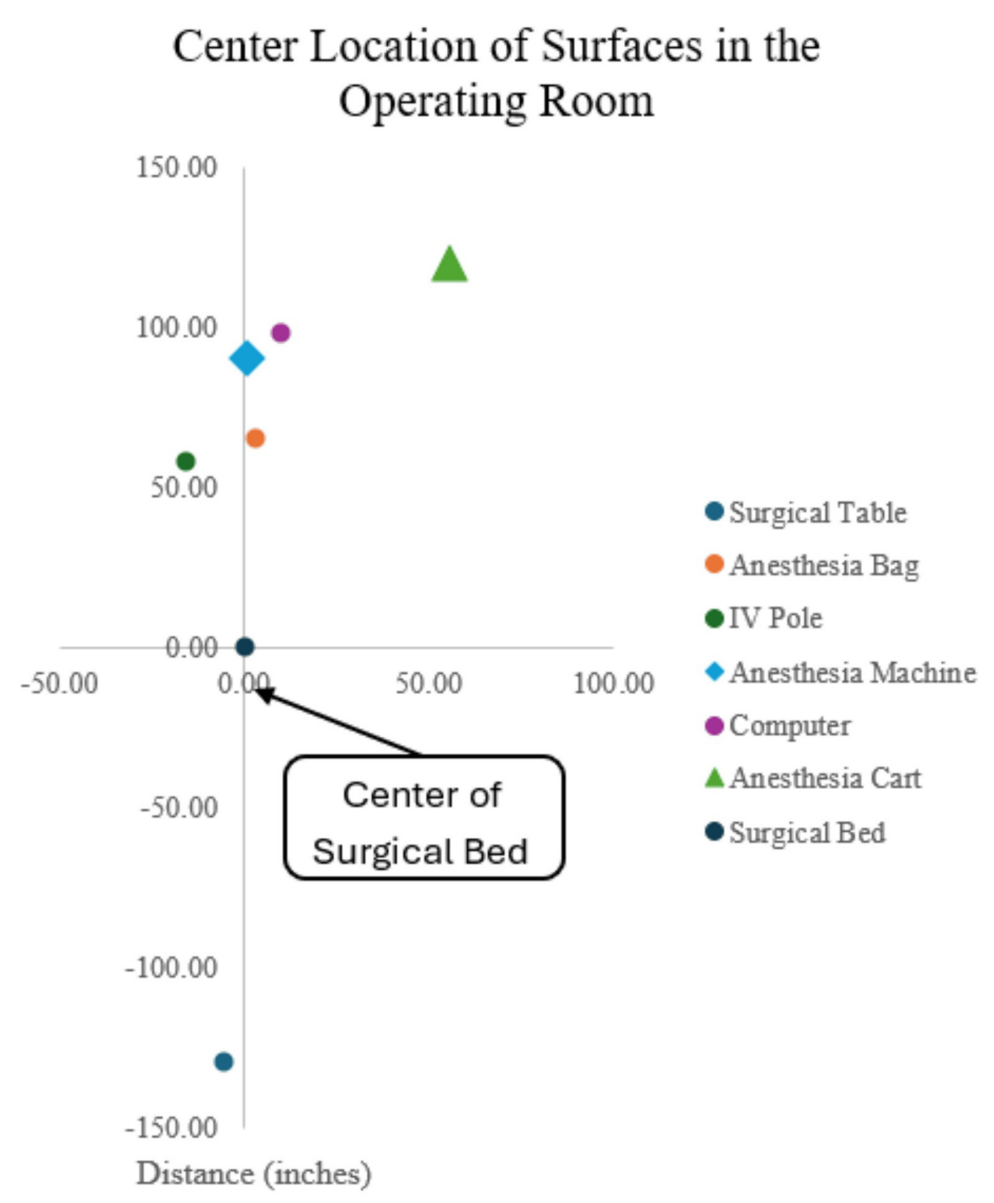
Typical location of operating room equipment at the end of terminal cleaning by axes (X (horizontal), Y (vertical), and Z (looking at the floor as a piece of paper, from the floor up) where distance (inches) is respective to the center of the surgical bed). Data were processed and visualized using Microsoft Excel (Microsoft Corp., Redmond, WA, USA).

This information was used to ensure that device-to-surface distance and orientation were standardized across the equipment evaluated (Figure 1).

As shown, the radiometer representing the anesthesia machine was placed at approximately 90 inches from the floor, while the radiometer on the anesthesia supply cart was placed at approximately 120 inches from the floor. The height of the cart, thereby, represents what is typical clinically, which is higher than previously evaluated [12-15] and includes a vertical lip that rises above the horizontal surface, thereby allowing an assessment of the impact of shadowing on device performance.

Radiometer positioning

A radiometer calibrated to capture the germicidal spectrum of irradiation via pulsed Xenon or UV-C (International Light Technologies, model ILT1270) was positioned horizontally to the emitters on the anesthesia machine tray, on the anesthesia cart, and outside of the window of the operating room at the distance from the center emitter specified by Figure 1. 

Ozone detection

A portable ozone gas detector (Vislone, Tangxia, China) equipped with an electrochemical O₃ sensor was utilized. The instrument has a measurement range of 0-50 ppm, a resolution of 0.01 ppm, and a response time of approximately 30 seconds. The manufacturer-specified accuracy is ±3-5% of full scale. The detector was zeroed prior to measurements.

The detector was placed on the top of the anesthesia machine, and the maximum reading was recorded following the treatment period. This position was chosen because it was close to the oxygen source, which was left on.

Use of UV-C equipment according to manufacturer recommendations

The Xenex machine (360-degree, single-emitter, pulsed Xenon) was placed in the center of the anesthesia workspace and ran for its maximum manufacturer-specified five-minute treatment cycle. 

For the Helios system (three emitters, triangular configuration, UV-C), one emitter was centered at the head of the surgical bed and one on each side of the head of the surgical bed. The allowed treatment time was up to 30 minutes or until 27 mJ/cm^2^ was reached for the radiometers on the anesthesia machine tray and cart. 

The TruD machine (one emitter rotating 360 degrees, UV-C) was placed in the center of the anesthesia workspace. The treatment time was up to 30 minutes or until 27 mJ/cm^2^ was reached for the radiometers on the anesthesia machine tray and cart. 

The Xenex machine allowed a treatment period of five minutes (the cycle time cannot be increased). The other devices were limited to 30 minutes to represent typical use [16]. All treatment replications were independent. The maximum irradiation dose for each radiometer was recorded at the end of the treatment cycle for each device.

Primary outcome

The primary outcomes were the irradiation doses recorded from radiometers that were positioned horizontally on both the anesthesia machine and cart (Figure 1) following treatment exposures. 

Secondary outcomes

Equipment setup and retrieval times, ozone generation, and inadvertent UV-C emittance through glass on the operating room door.

Sample size and power

The rationale for treatment times involving the delivery of the minimally effective dose for *S. aureus* attenuation on the anesthesia machine is that *S. aureus* is the sentinel pathogen for monitoring transmission among anesthesia workspace reservoirs to reduce SSIs [5,6,7,17]. *S. aureus* pathogens are tightly associated with SSIs [5-7] and the longitudinal spread of antibiotic resistance between surgical cases on different dates [10], while *Enterococcus *and Gram-negative spp. are not associated with infection or transmission of antibiotic resistance [18]. Furthermore, while *S. aureus* is frequently isolated among all anesthesia workspace reservoirs proven to be associated with transmission and SSI development, Gram-negative spp. are rarely isolated from the environment, and *Enterococcus* spp. are rarely isolated from intravascular devices; thus, both Gram-negative and *Enterococcus* spp. are unsuitable for monitoring [18].

To choose the sample size, we expected mean treatment times of approximately 18.3 minutes with a coefficient of variation of 19.6% [16]. Taking 18.3 minutes x 0.196 gave our expected 3.59 minutes.

Our primary comparisons were the assessment of the dose adequacy for biological effect. There were three machines compared pairwise, and P < 0.05 was treated as statistically significant, which was 0.0167 with Bonferroni adjustment for the three comparisons. For 90% statistical power from Stata statistical software (version 18.5, StataCorp LLC., College Station, TX), power twomeans 18.3, diff(5) sd(3.59) alpha(0.0167) power(0.9). Thus, N=16 per group was planned.

Statistical analysis

Given that these were independent replications, satisfying or not the criteria of at least 27 mJ/cm^2^ for both anesthesia machine and cart were compared using three pairwise Fisher exact tests, reported after Bonferroni adjustment for the three pairwise comparisons.

## Results

Xenex achieved 27 mJ/cm^2^ at the anesthesia machine for 1/16 trials and on the anesthesia cart for 0/16. TruD achieved a total of 27 mJ/cm^2^ at the anesthesia machine for 16/16 trials by five minutes and at the anesthesia cart for 0/16 trials by 30 minutes. Helios achieved the goal of 27 mJ/cm^2^ at the anesthesia machine for 4/8 by five minutes and 8/8 by a mean (standard deviation) of 16.2 (3.10) minutes, the dose thereby ranging from 13.1 to 19.3 minutes, and at the anesthesia cart for 0/8 by five minutes and 8/8 by 16.2 (3.10) minutes. Given the overall results of 0/16, 0/16, and 8/8, we stopped the trial. Pairwise comparisons showed that Helios performed significantly better than both other devices (adjusted P < 0.0001 for each comparison).

The time and maximum irradiation doses achieved for each of the three devices when used according to manufacturer recommendations, with a five-minute treatment time for Xenex and up to 30 minutes for Tru-D and Helios, are shown in Table 2.

**Table 2:** Cumulative irradiation doses (mean and standard deviation) for each emitter device when used according to manufacturer recommendations up to 30 minutes total.

	Cumulative irradiation dose (mJ/cm^2^)			
	Anesthesia machine		Anesthesia cart	
	Mean	SD	Mean	SD
Xenex (5 minutes)	30.97	4.84	4.05	1.37
TruD (30 minutes)	459.68	20.20	10.22	0.10
Helios (30 minutes)	113.04	49.52	27.11	0.32

The maximum irradiation doses for each of the three devices when employed for five minutes are shown in Table 3.

**Table 3:** Cumulative irradiation doses (mean and standard deviation) for each emitter device when employed for five minutes.

	Cumulative Irradiation Dose (mJ/cm^2^)			
	Anesthesia Machine		Anesthesia Cart	
	Mean	SD	Mean	SD
Xenex	30.97	4.84	4.05	1.37
TruD	74.34	6.08	1.61	0.04
Helios	27.55	9.88	7.31	2.09

The times required for setup/retrieval, ozone generation, and UV-C irradiation delivered to the radiometer positioned outside of the operating room window are shown in Table 4.

**Table 4:** Time required for setup/retrieval (minutes), ozone generation (parts per million, ppm), and UV-C irradiation dose (mJ/cm2) delivered to the radiometer positioned outside of the operating room for each emitter device.

	Setup time (minutes)		Ozone generation (ppm)		Irradiation (mJ/cm^2^)	
	Mean	SD	Mean	SD	Mean	SD
^Xenex^	3.76	0.28	0.10	0.0417	1.04	0.13
^TruD^	2.97	0.33	0.01	0.0058	0.25	0.01
^Helios^	3.26	0.58	0.02	0.0053	0.57	0.24

## Discussion

SSIs are a persistent issue associated with increased patient harm and healthcare costs [1-4]. Implementation of a multifaceted infection control program in the anesthesia workspace can generate substantial reductions in pathogen transmission and subsequent SSI development [5-7]. An important component of the multifaceted approach is environmental cleaning [8]. *S. aureus* MLST is a particularly pathogenic strain that is frequently transmitted among anesthesia workspace reservoirs despite usual cleaning procedures [9-11]. Recent work has begun to evaluate innovative cleaning strategies, including augmentation of surface disinfection cleaning with UV-C [12-15]. In this study, we primarily aimed to compare the time to delivery of the minimally effective dose to surfaces oriented horizontally to UV-C emitters at standardized distances and following manufacturer recommendations to provide strategic guidance for routine use of widely available UV-C equipment in the United States. In this study of widely available triangular and single emitter devices, we learned that the triangular configuration delivered the required dose to both target surfaces in 13-19 minutes. This was not true for the two single-emitter devices evaluated.

We have previously identified the target surfaces, sentinel pathogen, minimally effective dose, effective emitter configuration for UV-C treatment of the anesthesia workspace, and the time required for delivery of the minimally effective UV-C dose to surfaces oriented vertically to UV-C emitters [5-18]. In this study, we extend that knowledge to the assessment of the time required for delivery of the minimally effective dose for attenuation of MLST 5 on horizontal surfaces. We assessed up to 30 minutes, given typical turnover times [16]. We found that the Xenex machine when used according to manufacturer recommendations failed to deliver the minimally effective dose to either of the target surfaces, that the TruD device failed to deliver the required dose to the anesthesia cart, and that the Helios device, the configuration specifically designed to combat barriers to UV-C irradiation delivery, reliably delivered the minimally effective dose to both target surfaces within the 30-minute period. Thus, the single emitter devices were ineffective at dose delivery due to the inability to combat shadowing. As such, if using the single emitter devices evaluated in this study to treat the anesthesia workspace, they should be used in conjunction with dosimeters to ensure delivery of the minimally effective dose, and the dosimeters should be positioned at the distances and heights as described in this study (Table 1).

These results support the premise that one needs to understand how to utilize a technology in the clinical arena before evaluating the impact on SSI prevention. As the environment is one of the four necessary pillars of infection control, the bar is already quite high to show a difference in SSIs with a single intervention. Multifaceted interventions are superior to single interventions for SSI prevention involving the anesthesia workspace [19]. Showing an effect would even be less likely if the UV-C dose required for attenuation of the sentinel pathogen is not reliably delivered. The future role of UV-C [20] will depend on well-designed studies that account for the lesser impact of a single intervention in sample size calculations and involve optimized use of a device for attenuation of sentinel pathogens in the target environment.

We also assessed other factors such as the time required for setup/retrieval, ozone generation, and inadvertent UV-C exposure, as these are practical and safety considerations important for widespread adoption. While it may be contrary to widespread belief, the setup of three towers for the Helios configuration takes no longer than the setup of single emitters. This is because the three emitters are connected as one at the point of retrieval and setup. Thus, the setup period for the three devices consistently is around three minutes. The Xenex device produced the highest amount of ozone, though the threshold did not reach levels that would be thought to be associated with respiratory complications [21]. The recorded, inadvertent UV-C exposure cumulative for the study period was negligible for all three devices, where over 6 mJ/cm^2 ^in an eight-hour period is thought to be an occupational hazard [22].

This study may have been limited by the use of radiometers calibrated to the germicidal irradiation spectrum. However, our goal was to assess what is known as the germicidal spectrum, not theoretical combinations of wavelengths that may or may not have additive properties. In this way, we provided as best we could a head-to-head comparison. We further bolstered this approach by standardizing distances of emitters from target equipment and radiometers from emitters based on a pilot analysis of what are typical positions for equipment in the operating room after terminal cleaning. We also assessed only eight trials in the Helios group; however, further testing was not indicated, given the reliable impact of the device, which clearly distinguished it from single-emitter devices, findings supported by prior work. We understand that this study lacked validation of pathogen attenuation. This is because we have already done this work, showing that if the minimal dose for achieving a 6-log reduction is delivered to *S. aureus* MLST 5, it is effective [12-15]. Furthermore, this is true for less pathogenic *S. aureus *strains, and *S. aureus *is the sentinel pathogen that when transmission is attenuated, SSIs fall [12-15,17,18]. Of note, the 30 minutes was deliberately the maximum time used in many hundreds of operating rooms nationwide [16]. If a site wants to shorten the time of the UV-C to address between-case cleaning [23], what is needed is the use of the triangular configuration evaluated in this study (Helios), treatment of 13-19 minutes, following initial debulking of environmental contamination that does not require chemicals [15,24]. The effectiveness of the cleaning should be monitored [5-7], especially if alternative devices (Xenex, Tru-D) are used. If other pathogens, such as more pathogenic Gram-negative organisms, are identified in monitoring as an improvement target [25], treatment times will need to be adjusted. Such work is pending.

## Conclusions

We compared three UV-C devices in this study regarding the time required for delivery of the minimally effective dose required for attenuation of the more pathogenic *S. aureus* MLST 5 strain to horizontal surfaces. This was done to facilitate strategic implementation of available devices to achieve the desired aim. The triangular configuration achieved the target irradiation dose of 27.01 ± 0.15 mJ/cm^2 ^on both surfaces within the study conditions, while each of the single-emitter devices did not consistently reach the threshold on the anesthesia cart. These data support the impact of emitter configuration on dose delivery. If using the triangular configuration for *S. aureus* attenuation, a treatment time of 13.1-19.3 minutes is indicated for delivery of the minimally effective dose of 27.01 ± 0.15 mJ/cm^2^ to horizontal surfaces.
